# Two-Stage Surgery in Patients with Diffuse Glioma—Indications, Implications and Outcome

**DOI:** 10.3390/cancers18050722

**Published:** 2026-02-24

**Authors:** Sebastian Jeising, Johannes Reinken, Marion Rapp, Michael Sabel, Franziska Staub-Bartelt

**Affiliations:** 1 Institute of Neuropathology, Heinrich-Heine-University Düsseldorf, 40225 Düsseldorf, Germany; 2 Department of Neurosurgery, Uniklinikum Erlangen, Friedrich-Alexander-Universität Erlangen-Nürnberg, 91054 Erlangen, Germany; 3 Medical Faculty, Heinrich-Heine-University, 40225 Düsseldorf, Germany; 4 Brain Cancer Centre, Beta Clinic Bonn, 53227 Bonn, Germany; 5 Department of Neurosurgery, University Hospital Giessen, 35392 Giessen, Germany

**Keywords:** glioma surgery, two-staged surgery, diffuse glioma, advances in glioma surgery, extent of resection

## Abstract

Introduction: Significant studies have substantiated the evidence for complete resection of intrinsic brain tumours in recent years. However, achieving this through a single surgery is not always possible due to tumour localisation in eloquent areas. Therefore, the present analysis aimed to evaluate surgical outcomes in a cohort of patients undergoing planned two-stage glioma surgery. Methods: Patients who underwent surgery for diffusely infiltrating brain tumours between 2013 and 2023 at the Department of Neurosurgery at Düsseldorf University Hospital were screened for undergoing two-stage surgery, defined by *a priori*-considered surgical re-intervention up to 6 weeks after the initial surgery. Results: Of 1558 patients with glioma, 447 underwent multiple surgeries, of whom 36 underwent planned two-stage surgery during the course of their disease. Two-stage surgery was performed mostly as glioma surgery at first diagnosis (75%). The mean time between the first and second surgery was 11.67 days (±7.59). Two-stage surgery was performed due to various reasons, mostly in localisations that required multifocal approaches (47.2%), due to non-compliance during initial awake surgery (30.6%), or cases with primary debulking for subsequent awake-surgery approaches (22.2%). Tumours were mainly located in the left hemisphere (50%) (right hemisphere 25%, or bilateral 25%) and motor- or speech-eloquent in 61.11%. Tumours were 72.2% IDH-wildtype. An intended complete resection result was achieved in 58.88% after the second surgery, changing from 93.94% submaximal resection to 58.88% supramaximal and maximal resection after the second surgery. Second surgery significantly reduced residual tumour volume of both T1-CE (Wilcoxon signed-rank test, Z = −4.62, *p* < 0.001) and T2-nCE (Z = −4.62, *p* < 0.001). In contrast, functional (KPS: Z = −0.93, *p* = 0.350) and neurological status (NIHSS: Z = −0.89, *p* = 0.372) did not significantly change. Perioperative complications of the second surgery occurred in nine (25%) cases, requiring surgical intervention under general anaesthesia or ICU treatment (Clavien–Dindo grade IIIb/IV) in six (16.67%) cases. Conclusion: Planned two-stage surgery was mostly performed as a surgical strategy in eloquent locations to achieve supramaximal or maximal resection. A two-staged surgery significantly extended resection results without neurological and functional deterioration. Despite relevant complication rates, primary debulking followed by staged resection as well as two-staged multifocal approaches may yield a favourable risk–benefit profile.

## Simple Summary

Maximal safe tumour resection is a key prognostic factor in patients with diffuse gliomas, but complete resection is often limited by tumour location in eloquent brain areas. In this retrospective single-centre study, we analysed outcomes of patients undergoing a planned two-stage surgical approach for diffuse gliomas. Two-stage surgery was mainly used for eloquent or multifocal tumours, non-compliance during awake surgery, or as a primary debulking strategy. The second surgery significantly reduced residual tumour volume and increased rates of maximal or supramaximal resection without relevant deterioration of neurological or functional status. Although complication rates were not negligible, planned two-stage surgery appears to be a feasible strategy in selected patients to extend the extent of resection while preserving neurological function.

## 1. Introduction

Complete tumour resection has emerged as a cornerstone in the management of diffusely infiltrating gliomas, with multiple studies demonstrating that a greater extent of resection is associated with improved progression-free (PFS) and overall survival (OS). For instance, contrast-enhancing (CE) tissue removal in glioblastoma has been linked to longer progression-free survival [1,2], and recent data suggest that resection of non-contrast-enhancing (nCE) tumour beyond a volume threshold of <5 cm^3^ further improves survival outcomes [3,4,5].

While techniques such as awake mapping [6,7,8], intraoperative neurophysiological monitoring [9], and advanced intraoperative visualisation (e.g., fluorescence-guided surgery or intraoperative ultrasound) have been developed to navigate the trade-off between oncological benefit and functional preservation [10], some tumours remain incompletely resectable in a single procedure. This is due to factors such as multifocal localisation, patient cooperation during awake surgery, or preoperative impairment of neurological status due to tumour mass effects. In these settings, a two-staged surgical strategy may extend resection results while preserving neurological performance.

In oncologic surgery, staged resection has been recognised as a strategic approach to balance maximal tumour removal with procedural safety. The concept was first formalised in hepatic surgery, where two-stage hepatectomy was introduced as a planned strategy to achieve complete resection in patients with initially unresectable bilobar liver metastases while preserving sufficient functional parenchyma [11]. Beyond illustrative cases [12,13], there is no evidence for *a priori*-staged glioma surgery, with no prospective comparative cohorts to date.

Building on the growing interest in functional and neurological outcomes in neuro-oncology, this study conducts a single-centre retrospective evaluation of surgical outcomes in a cohort of patients with planned two-staged glioma surgery. Therefore, we analysed different indications for this strategy, measured changes in residual tumour volumes, assessed perioperative complications and determined functional and neurological outcomes over the course of surgical procedures.

## 2. Methods

### 2.1. Patients

Patients undergoing diffuse glioma surgery between 2013 and 2023 at the Department of Neurosurgery, University Hospital Düsseldorf, were screened for *a priori* consideration of surgical re-intervention up to 6 weeks after the initial surgery, naturally excluding postoperative complications requiring surgery. The complete institutional database comprised 1558 glioma patients during this period, of which 447 patients underwent at least one re-operation. In total, 46 patients had unplanned second-look surgery due to unexpected residual tumour and are not discussed in this manuscript. Within this cohort, 36 patients fulfilled criteria for two-stage surgery.

### 2.2. Clinical Data

Sociodemographic data, neuropathological results, information on medical/surgical history, if applicable, adjuvant therapy, and surgical details—such as use of intraoperative neurophysiological monitoring (IONM), brain mapping techniques and fluorescence-guided surgery using 5-aminolevulinic acid (5-ALA)—were obtained from the local patient administration system (CGM Medico, CompuGroup Medical, Koblenz, Germany).

### 2.3. Functional and Neurological Outcome Data

KPS and NIHSS were retrospectively noted from clinical recordings at the three timepoints of interest for this study, including initial status before the first surgery attempt, preoperatively before the second surgical attempt and postoperatively after the two-staged attempt. In order to evaluate occurring complications, the Clavien–Dindo Classification [14] was used.

### 2.4. Neuropathological Classification

Neuropathological diagnosis and WHO grading were performed according to the World Health Organisation (WHO) classification of tumours of the central nervous system, valid at the time of surgery [15,16,17].

### 2.5. Extent of Resection

Assessment of the EOR was based on early postoperative MRI performed within 72 h after surgery. The imaging protocol included gadolinium contrast-enhanced T1-weighted (T1-CE) sequences to delineate enhancing tumour, T2/FLAIR for non-enhancing (T2-nCE) infiltrative components, and diffusion-weighted imaging to assess perioperative ischemia. Tumour volumetry was conducted using the Brainlab Elements platform (Brainlab GmbH, Munich, Germany). The SmartBrush tool enabled semi-automated region-growing segmentation of pre- and postoperative tumour compartments (CE and nCE) and resection cavities. Residual tumour volume was measured in cubic centimetres for CE and nCE compartments. In addition to raw volumetric measurements, resections were classified according to the RANO resect classification system, validated as a prognostic marker in both primary and recurrent glioblastoma: RANO class 1: “supramaximal CE resection” (0 cm^3^ CE + ≤5 cm^3^ nCE), class 2: “maximal CE resection” (2A: “complete CE resection” (0 cm^3^ CE + >5 cm^3^ nCE); 2B: “near total CE resection” (≤1 cm^3^ CE)), class 3: “submaximal CE resection” (3A: “subtotal CE resection” (≤5 cm^3^ CE); 3B: “partial CE resection” (>5 cm^3^ CE)), class 4: “biopsy” (no reduction in tumour volume) [4,18].

### 2.6. Statistical Analyses

All statistical analyses were performed using IBM SPSS Statistics (Version 29.0; IBM Corp., Armonk, NY, USA). Quantitative data were assessed for normality using the Shapiro–Wilk test. As none of the continuous variables, including tumour volumetry, functional and neurological scores, and time intervals, showed a normal distribution, all analyses were conducted using non-parametric statistical tests. Paired continuous and ordinal variables (e.g., pre- and postoperative tumour volumes, Karnofsky Performance Status, and NIHSS) were compared using the Wilcoxon signed-rank test, while independent continuous variables were analysed using the Mann–Whitney U test. Categorical variables were compared using the χ^2^ test or Fisher’s exact test, as appropriate.

Categorical variables are expressed as counts and percentages. The percentages refer to the complete data points available in each case. All statistical tests were two-tailed, and *p* < 0.05 was considered statistically significant.

All graphics (e.g., violin and scatter plots) were generated in Python (Version 3.9, Google Colab environment) using matplotlib and seaborn. Sankey diagrams illustrating categorical data transitions were created with SankeyMATIC (www.sankeymatic.com), and schematic illustrations were designed with BioRender.com.

### 2.7. Ethics Approval

This retrospective study was approved by the ethics committee of Heinrich Heine University Düsseldorf (Study ID: 2025-3212).

## 3. Results

### 3.1. Overview

**Figure 1:**
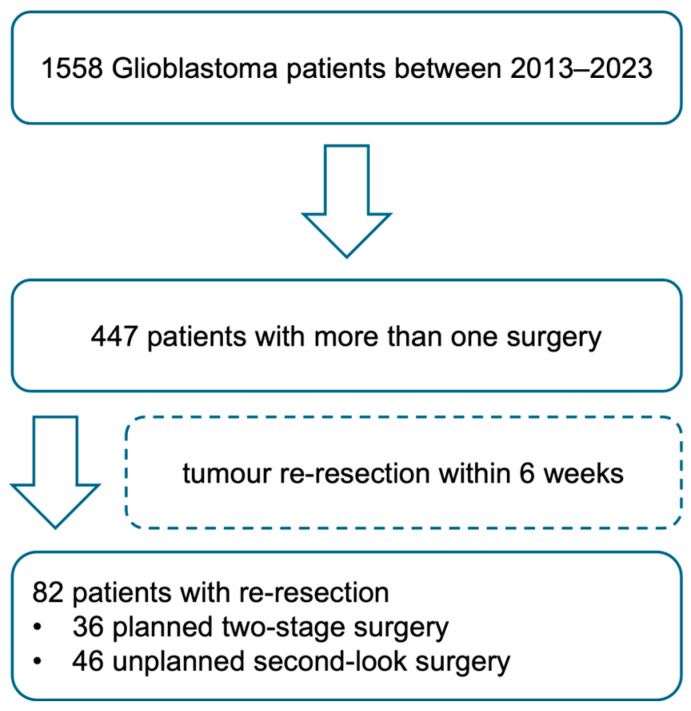
Flowchart of the patient selection process.

Between 2013 and 2023, 1558 patients were diagnosed with glioma after brain tumour surgery at the Department of Neurosurgery at Düsseldorf University Hospital. A total of 447 patients underwent more than one surgery during the course of their disease. In 36 cases, the neurosurgical strategy comprised *a priori*-considered two-stage surgery. Figure 1 summarises patient selection within a flowchart

All resections followed contemporary principles of glioma surgery, aiming for maximal safe resection while preserving neurological function. Surgical strategy was adapted to tumour location, relation to eloquent structures, and patient factors. Standard adjuncts included neuronavigation, IONM, awake cortical and subcortical mapping when indicated, fluorescence-guided surgery with 5-ALA, and intraoperative ultrasound (ioUS). Adjuvant therapies were administered according to the EANO guidelines on the diagnosis and treatment of diffuse gliomas of adulthood, valid at the time [19,20,21].

**Figure 2:**
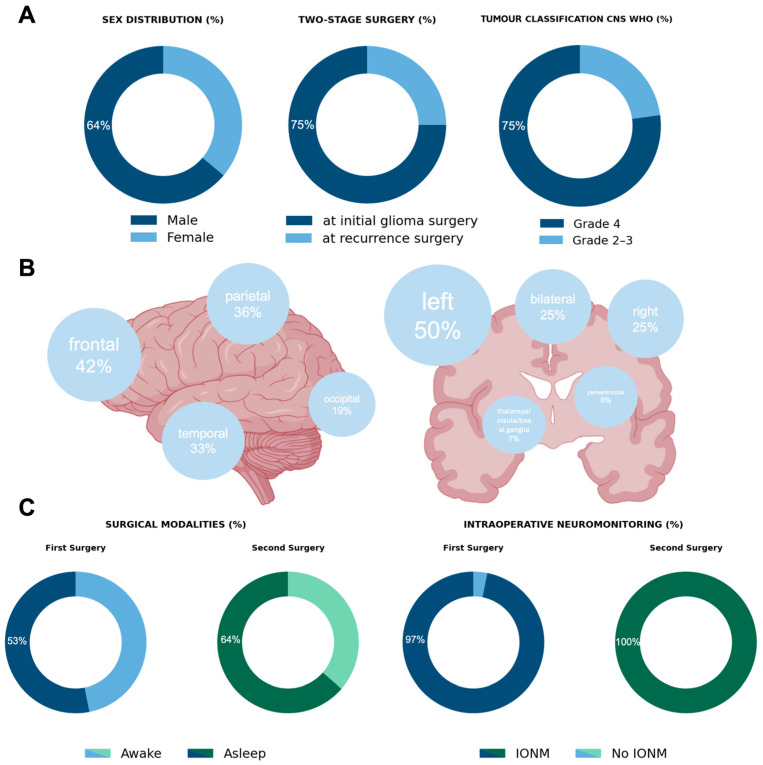
Characteristics of the patient cohort. (**A**): Sex distribution within the cohort, time point of surgery (initial vs. recurrence), and tumour classification. (**B**): Tumour localisation in the included patients. (**C**): Surgical strategy including use of awake surgery and intraoperative neurophysiological monitoring. Figure 2B was created using BioRender.com.

Two-stage surgery was performed mostly as glioma surgery at first diagnosis (75%). The mean time between the first and second surgery was 11.67 days (±7.59). In this cohort, 63.89% of the patients were male with a mean age of 49.08 years (SD ± 13.94) and a median preoperative KPS of 90 (range 50–100) and NIHSS of 0 (range 0–7). Median Charlson comorbidity index was 3 (range 2–6). Mean CE tumour volume before first surgery was 28.78 cm^3^ (±5.25) and mean nCE tumour volume was 12.96 cm^3^ (±3.17). Tumours were 72.2% IDH-wildtype and classified according to the CNS WHO classification, used at the time of diagnosis, as WHO grade 4 in 75% and 2–3 in 25%. Within the grade 4 tumours, there were 26 glioblastomas and one IDH-mutant astrocytoma. Of the glioblastomas, MGMT promoter was methylated in 50%. There were two grade 2 and six grade 3 tumours, all with IDH mutations, and none with 1p/19q-codeletion (Figure 2A).

Tumours were mainly located in the left hemisphere (50% left hemisphere, right hemisphere 25%, or bilateral 25%) and motor- or speech-eloquent in 61.11% (speech-eloquent in 15 cases, motor-eloquent in six cases and combined in one case) (Figure 2B).

The subgroup of glioblastoma patients who underwent a two-stage surgical approach, as primary resection for glioblastoma, comprised 20 patients. Of them, 14 patients received standard-of-care radio-chemotherapy with temozolomide according to the Stupp protocol [22], and 3 patients were administered the Herrlinger protocol [23], adding chemotherapy with lomustine (CCNU). The mean interval between the first surgery and initiation of radio-chemotherapy was 37.5 days (range: 29–66).

### 3.2. Surgical Strategy

Two-stage surgery was performed due to various reasons. The most common reasons for conducting a second surgery were localisations that required multifocal approaches (47.2%), non-compliance during initial awake surgery (30.6%) or cases in which patients underwent primary debulking for subsequent surgery under awake or extended electrophysiological testing (22.2%). Surgical strategy comprised awake surgery in 46.88%, intraoperative neuromonitoring in 96.88% and fluorescence-guided surgery using 5-ALA in 87.5% (*n* = 28, after administration in 30 patients) during the first surgery. During the second surgery, an awake procedure was performed in 36.36%, while intraoperative neuromonitoring and fluorescence-guided surgery using 5-ALA were used in 100% and 78.79% of procedures respectively (5 ALA, *n* = 26, after administration in 26 patients) (Figure 2C). Resections were conducted by specially trained consultant neuro-oncological neurosurgeons.

### 3.3. Resection Results

**Figure 3:**
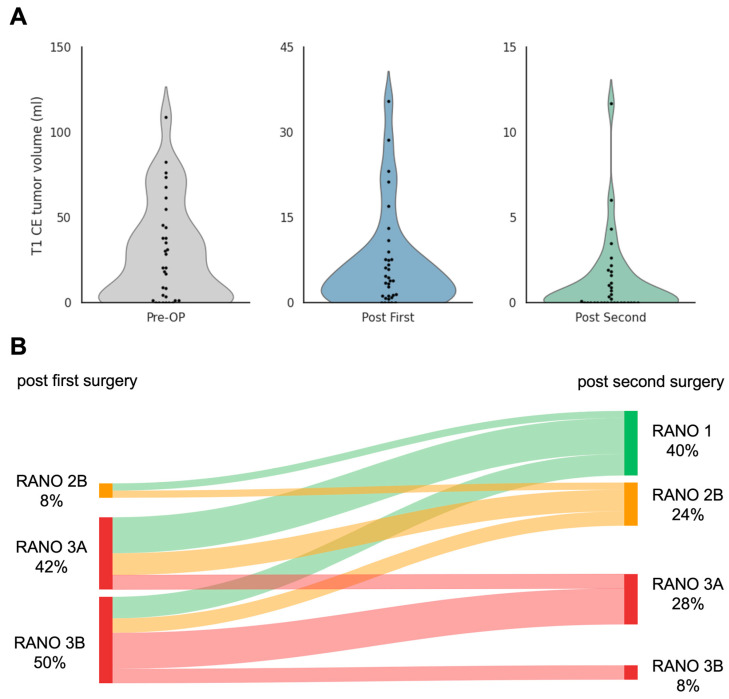
Tumour volumes and change in RANO class from first to second surgery. (**A**): Violin plots showing T1-weighted contrast-enhancing (T1-CE) tumour volumes preoperatively (Pre-OP), after the first surgery (Post First), and after the second surgery (Post Second). A significant stepwise reduction in residual enhancing tumour volume is observed after each resection. The black dots represent single patients. (**B**): Sankey diagram visualising within-patient transitions in RANO resection class between the immediate postoperative assessment after the first surgery (left; classes present: RANO 2B, 3A, 3B) and after the second surgery (right; classes: RANO 1, 2B, 3A, 3B). Ribbon width is proportional to the number of patients; colours correspond to the destination class. The plot demonstrates a net shift toward lower residual volume following the second surgery, with numerous patients improving from RANO 3A/3B to RANO 1 or RANO 2B, while a subset remains unchanged within higher classes. Abbreviation: RANO, Response Assessment in Neuro-Oncology (higher classes indicate greater residual (non-)contrast-enhancing tumour burden).

Resection results of tumours classified as glioblastoma (*n* = 26) after the first surgical attempt corresponded to RANO resect class 2B in 8.33%, 3A in 41.67% and 3B in 50%. All other tumours (*n* = 9) had >1 cm^3^ residual tumour (T2-nCE). After the second surgical attempt, the intended complete resection result was achieved in 64%, changing EOR from 93.94% submaximal resection to 64% supramaximal and maximal resection. Second surgery significantly reduced residual tumour volume in both T1-CE (Wilcoxon signed-rank test, Z = −4.62, *p* < 0.001) and T2-nCE (Z = −4.62, *p* < 0.001). After the second surgery, resection results corresponded to RANO resect class 1 in 40%, 2B in 24%, 3A in 28% and 3B in 8% (Figure 3). Residual tumour volumes of all other tumours (*n* = 9) were >1 cm^3^ in five cases, <1 cm^3^ in three cases and complete resection in one case.

### 3.4. Outcome

**Figure 4:**
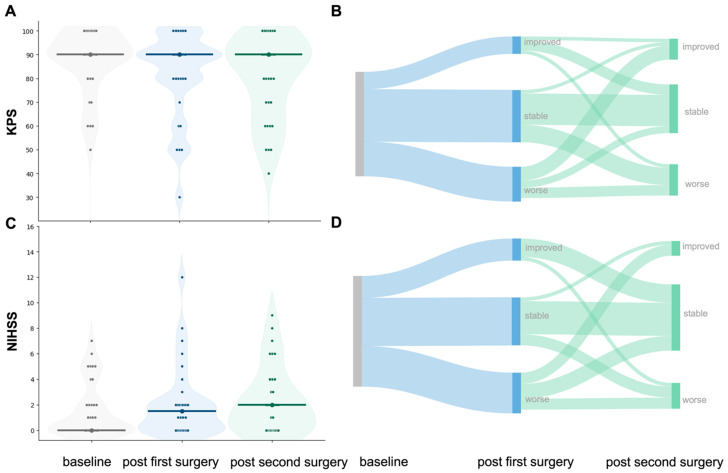
Functional and neurological performance over the course of surgeries. (**A**): Violin plots depict the distribution of KPS at three time points: preoperative baseline, post first, and post second surgery. Individual patient values are overlaid; central markers indicate summary tendency (median and spread). (**B**): Corresponding Sankey diagram showing within-patient transitions in relative KPS (improved, stable, worse) from baseline to post first surgery to post second-look surgery. Ribbon width is proportional to the number of patients following each trajectory. (**C**): Violin plots for the National Institutes of Health Stroke Scale (NIHSS) at the same time points, with individual observations overlaid. (**D**): Corresponding Sankey diagram for relative NIHSS changes across the same time points.

Median KPS was 90 (range 30–100) and median NIHSS was 1.5 (0–12) after the first surgery, and 90 (KPS, range 40–100) and 2 (NIHSS 0–9), respectively, after the second surgery (Figure 4A–D). Yet, functional (KPS: Z = −0.93, *p* = 0.350) and neurological status (NIHSS: Z = −0.89, *p* = 0.372) did not significantly change between the postoperative assessments after the first and second surgical attempt.

Perioperative complications in connection to the second surgery occurred in 14 cases (38.89%), requiring surgical intervention under general anaesthesia or ICU treatment (Clavien–Dindo grade IIIb/IV) in six cases (16.67%), with one wound infection, two cases of hydrocephalus and three CSF leaks. The 30-day perioperative mortality was 0%.

After the second surgery, twelve patients still had submaximal resection results (RANO class 3). Of those, four additionally experienced worsening of functional and neurological status with new deficits after the second surgery. Statistical analysis did not identify significant risk factors for this constellation of submaximal resection with neurological and functional deterioration. Functional and neurological status did not reach statistical significance in difference after first or second surgery compared to the whole cohort (median KPS after first surgery: 90 (60–100), after second surgery: 75 (60–90); median NIHSS after first surgery: 1 (0–6), after second surgery: 4 (1–7)). Only one of these patients had a better resection result after the second surgery, changing from RANO class 3B to 3A.

### 3.5. Subgroup Assessment

#### 3.5.1. Multifocal Approaches

Mean tumour volume in the subset of multifocal approaches (*n* = 17) was 27.6 cm^3^ (±7.5) for T1-CE and 13.3 cm^3^ (±5.3) for T2-nCE. Localisation was mostly bilateral in 52.94% of cases (23.53% right-hemispheric, 23.53% left-hemispheric). Tumour localisation was motor/speech-eloquent in 52.94%.

After the first surgery, residual volumes were reduced to 7.2 cm^3^ CE parts (±2.2) and 4.0 cm^3^ nCE parts (±1.8), respectively, and further decreased after the second surgery to 1.6 cm^3^ (±0.8) and 0.76 cm^3^ (±0.34). The intended complete resection result was achieved in 56.25% due to the second surgery, changing from 86.67% submaximal resection after the first surgery to 56.25% supramaximal and maximal resection after the second surgery. RANO resect class 1 was achieved in 50% (*n* = 6), 2B in 8.34% (*n* = 1), 3A in 25% (*n* = 3) and 3B in 25% (*n* = 2).

**Figure 5:**
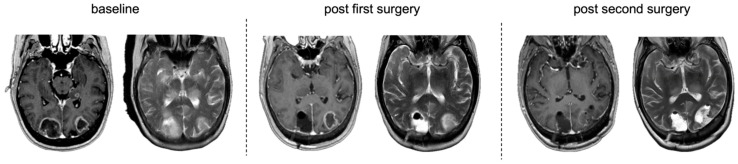
MRI from a representative case with multifocal approaches and supramaximal resection after two-stage surgery. From left to right. T1-CE and T2-nCE baseline to post second surgery. Postoperative MRI demonstrating T1-CE as well as T2-nCE residual tumour in the left occipital lobe. MRI after the second surgery shows no residual tumour.

Complete resection was achieved in one LGG; one patient showed residual tumour volume with <1 cm^3^ and two patients with >1 cm^3^ residual volume. Second surgery also significantly reduced residual tumour volume in this subcohort, for both T1-CE (Wilcoxon signed-rank test, Z = −3.30, *p* < 0.001) and T2-nCE (Z = −2.93, *p* = 0.003), while functional and neurological status remained stable (KPS: Z = −1.28, *p* = 0.2; NIHSS: Z = −0.68, *p* = 0.498). New neurological deficits occurred in 41.2% (*n* = 7) of patients after the first surgery and in 11.67% (*n* = 2) (one motor, one motor and speech deficit and reduced vigilance) after the second surgery. Yet there was no significant change in functional and neurological status between postoperative assessments after the first and second surgery (KPS: Z = −1.282, *p* = 0.2; NIHSS: Z= −0.677, *p* = 0.498). Peri- and postoperative complications of the second surgery occurred in seven (46.67%) cases, requiring surgical intervention under general anaesthesia or ICU treatment (Clavien–Dindo grade IIIb or IV) in three cases, with one wound infection and two CSF leakages. Figure 5 provides an illustrated case of multifocal approaches included in the study.

#### 3.5.2. Non-Compliance at the First Surgery

**Figure 6:**
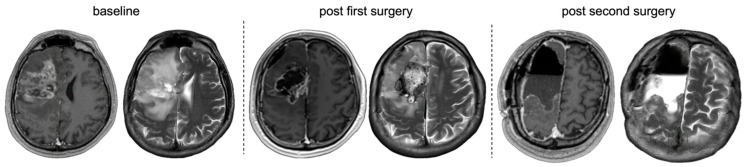
MRI from a representative case with non-compliance at first surgery and supramaximal resection after two-stage surgery. From left to right. T1-CE and T2-nCE baseline to post second surgery. Postoperative MRI after first awake surgery, right frontal demonstrating T1-CE as well as T2-nCE residual tumour. MRI after the second surgery shows no residual T1-CE or T2-nCE tumour.

A decision for a two-staged surgical approach was made intraoperatively during the first surgery, aware of significant residual tumour volumes. Therefore, all patients in the subgroup of non-compliant patients during the first surgery (*n* = 11) had only submaximal resection. Tumour localisation was motor/speech-eloquent in 72.73%. Subsequent surgery (60% awake, 40% asleep) further reduced tumour volume, improving to 36.36% supramaximal and maximal resection after the second surgery. After the second surgery attempt, resection results of patients diagnosed with glioblastoma corresponded to RANO resect class 1 in 16.67%, 2 in 33.34% and 3A in 50%. Residual tumour volume within the IDH-mutant gliomas (*n* = 4) was >1 cm^3^ in three cases and <1 cm^3^ in one case. Residual tumour volume was significantly reduced in both T1-CE (Wilcoxon signed-rank test, Z = −2.52, *p* = 0.012) and T2-nCE (Z = −2.80, *p* = 0.005). Functional and neurological status again remained stable, with no significant changes in postoperative KPS (Z = −0.28, *p* = 0.783) or NIHSS (Z = −0.73, *p* = 0.465). New neurological deficits occurred in 18.18% (*n* = 2) after the first surgery and in 36.36% (*n* = 4) of cases (three motor, one motor and speech deficits) after the second surgery. Peri- and postoperative complications of the second surgery occurred in four (36.36%) cases, requiring surgical intervention under general anaesthesia (Clavien–Dindo grade IIIb) in one case with hydrocephalus. Figure 6 provides an illustrated case of an intraoperative non-compliant patient with consecutive two-staged surgery.

#### 3.5.3. Primary Debulking

**Figure 7:**
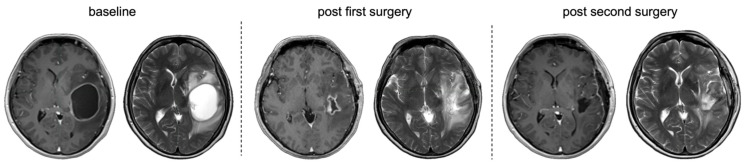
MRI from a representative case with primary debulking and supramaximal resection after two-stage surgery. From left to right. T1-CE and T2-nCE baseline to post second surgery. Postoperative MRI after the first surgery on a cystic left parieto-temporal lesion demonstrating residual T1-CE as well as T2-nCE residual tumour. MRI after the second surgery shows no residual tumour.

In the subset of a primary debulking approach (*n* = 8), mean preoperative CE tumour volume was 38.07 cm^3^ (±13.19) and nCE tumour volume was 9.54 cm^3^ (±4.26). Tumour localisation was motor/speech-eloquent in 62.5%. Compared to patients undergoing two-stage surgery for multifocal approaches or due to non-compliance, volumes were higher, but these differences did not reach statistical significance. Speech-eloquent tumours were more frequent (50% vs. 42.85%), while motor-eloquent tumours were less frequent (12.5% vs. 21.43%) compared to the entire cohort. Primary debulking was performed mostly asleep (83.34%) and reduced tumour volume to 8.22 cm^3^ (±3.97) CE and 2.14 cm^3^ (±0.71) nCE. Subsequent surgery (awake 28.57%, *n* = 2) further reduced tumour volume down to 0.40 cm^3^ (±0.24) CE and 0.34 cm^3^ (±0.21) nCE, changing from 100% submaximal resection to 87.5% supramaximal and maximal resection after the second surgery. After the second surgery, resection results corresponded to RANO resect class 1 in 42.85% (*n* = 3), 2B in 42.85% (*n* = 3) and 3A in 14.29% (*n* = 1). In one IDH-mutant glioma residual tumour, volume was <1 cm^3^. Residual tumour volume was significantly reduced in both T1-CE (Wilcoxon signed-rank test, Z = −2.37, *p* = 0.018) and T2-nCE (Z = −2.37, *p* = 0.018). Functional and neurological status remained stable, with no significant changes between postoperative assessments after the two measured timepoints (KPS: Z = −0.58, *p* = 0.564; NIHSS: Z = −0.45, *p* = 0.655). However, new neurological deficits occurred in 12.5% (*n* = 1) of patients after the first surgery and in 25% (*n* = 2) (one motor deficit and reduced vigilance, one vision/cranial nerve deficit) after the second surgery. Peri- and postoperative complications of the second surgery occurred in three (37.5%) cases, requiring surgical intervention under general anaesthesia (Clavien–Dindo grade IIIb) in two cases, one with hydrocephalus and one with CSF leakage. Figure 7 shows an illustrated case of primary tumour debulking before supramaximal resection in a planned two-staged approach.

A comprehensive overview of the study cohorts’ results is presented in Table 1.

## 4. Discussion

This study provides the first systematic evaluation of two-stage surgery in diffuse infiltrating gliomas. While complete or supramaximal resection has repeatedly been shown to correlate with improved survival [1,3,4,5], achieving this goal in tumours located within eloquent areas remains challenging. Our findings indicate that a staged approach can significantly increase the extent of resection without compromising functional or neurological outcomes, even in a population with a high proportion of eloquent tumours.

Patients in our cohort had a mean age of 49 years, a median preoperative KPS of 90, and an NIHSS of 0, although 50% of the tumours were located in the left hemisphere and more than 60% involved motor- or speech-eloquent regions. These baseline characteristics are similar to those in large extent-of-resection analyses, in which younger age and preserved performance status predicted the greatest oncological benefit from aggressive resection [3,24]. In our cohort, the surgical strategy remained consistent between the first and second surgeries, with awake mapping performed in approximately half of patients, intraoperative neuromonitoring in nearly all cases, and 5-ALA fluorescence guidance in about 80%. Achievements after the second procedure were therefore attributable not to technical escalation but to the deliberate choice to stage resection. These findings reinforce prior reports that intraoperative mapping, neurophysiological monitoring, and fluorescence guidance are essential to balance oncological benefit and functional preservation [9,10].

After the first surgery, almost all patients (93.9%) remained in a submaximal resection category. Following the second surgery, 64% achieved supramaximal or maximal resection, with significant volumetric reductions in both CE and nCE tumour. These results must be considered in light of the data from Molinaro et al. and Karschnia et al., which showed that the comprehensive removal of both compartments is independently associated with an improved survival rate. Importantly, functional outcomes remained stable between surgeries, with no significant changes in KPS or NIHSS, consistent with recent multicentre analyses demonstrating that complete resection can be achieved without deterioration in quality of life when modern intraoperative techniques are applied [25].

**Figure 8:**
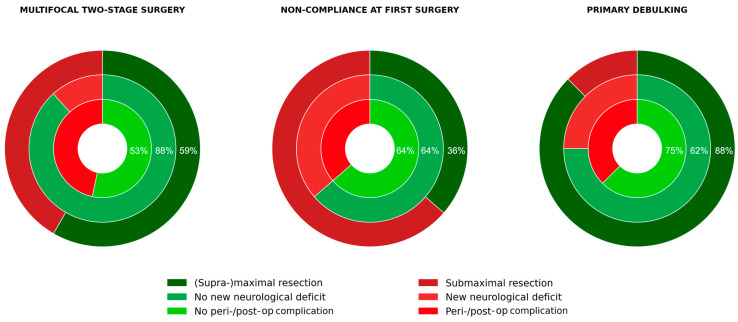
Risk–benefit illustration after the second surgery. Concentric ring charts illustrate key outcome parameters following two-stage surgery for diffuse infiltrating gliomas in different indication groups. Multifocal approaches: (Supra-)maximal resection was achieved in 58.3% of patients, with 88.3% remaining neurologically stable and peri-/postoperative complications occurring in 46.7%. Non-compliance at first surgery: (Supra-)maximal resection was achieved only in 36.36 of cases, with 63.6% of patients showing no new neurological deficits and 63.6% experiencing no complications. Primary debulking: (Supra-)maximal resection was achieved in 87.5% of patients, 75% remained neurologically stable, and 62.5% had an uneventful postoperative course.

However, complication rates after the second surgery were not negligible. Perioperative complications occurred in 25% and serious Clavien–Dindo grade IIIb/IV events in 16.7% of the patients. These figures are comparable to complication rates reported in another series of aggressive glioma surgery in eloquent locations [26]. Importantly, 30-day mortality was 0%, and the risk–benefit profile must be considered acceptable in light of the potential oncological gain. Subgroup analyses revealed that patients undergoing staged surgery after primary debulking achieved the most favourable balance, with 87.5% reaching supramaximal or maximal resection and stable neurological outcomes despite higher preoperative tumour volumes. Similarly, patients with multifocal gliomas achieved acceptable volumetric reductions (56.25% supramaximal and maximal resection) and had low rates of new neurological deficits and perioperative complications. In contrast, in cases of intraoperative noncompliance during awake surgery, only 36.36% reached supramaximal or maximal resection after the second procedure, and more than a third developed new deficits, raising concerns about the benefit of staging in this context (Figure 8). These differences highlight the importance of careful patient selection.

A further consideration in the neuro-oncological context is whether staging delays adjuvant therapy. In our series, the mean interval between the first surgery and initiation of radio-chemotherapy was 37.5 days, which did not critically prolong treatment initiation compared to benchmarks [27]. This finding suggests that a two-stage approach can be integrated into standard-of-care treatment pathways without oncological compromise.

Staged resection has been recognised as a strategic oncological approach to balance maximal tumour removal and tissue recovery in other-than-neuro-oncological contexts, for example, in hepatectomy [11]. Evidence on planned two-staged neuro-oncological resections in glioma resections has so far been limited to case reports. These include cyst decompression followed by resection [28], staged multilobe low-grade glioma resections [12], and reports demonstrating functional plasticity in low-grade gliomas [13]. Duffau and Taillandier have proposed a multistage approach over the years for low-grade gliomas, leveraging brain plasticity to optimise the extent of resection while preserving quality of life [29]. Our study evaluates two-staged surgery for rapidly growing high-grade gliomas, which do not respond sustainably to chemotherapy, showing that even short-interval staged resections can safely increase resection volumes without worsening functional outcomes.

## 5. Limitations

Nevertheless, the present work has several limitations. The retrospective single-centre design limits generalisability, and the small subgroup sizes preclude robust statistical analysis of risk factors for postoperative deterioration as well as survival analyses. Selection bias is inevitable, as staged resections were reserved for particularly complex cases. Finally, while short-term neurological and functional stability was demonstrated, long-term outcomes on survival and quality of life remain to be determined.

## 6. Conclusions

In conclusion, planned two-stage resection represents a feasible surgical strategy for complex gliomas in eloquent areas. It can significantly extend resection volumes without functional compromise in most patients, though complication rates remain relevant. Subgroup analyses suggest that primary debulking followed by staged resection as well as multifocal approaches may yield the most favourable risk–benefit profile, whereas non-compliant awake cases should be approached cautiously. Future prospective multicentre studies, ideally stratified by molecular subtype, are warranted to define selection criteria and clarify the long-term oncological impact of this approach.

## Figures and Tables

**Table 1:** Patients.

Patients (*n*)			36
Demographics			
Age (years, mean, SD)			49.08 ± 13.94
F:M ratio			1:1.77
Two-stage surgery (*n*)			
At initial glioma surgery			27
At recurrence surgery			9
Mean time between surgeries (days, mean, SD)			11.67 ± 7.59
Localisation (*n*)			
Left hemisphere			18
Right hemisphere			9
Frontal involvement			15
Parietal involvement			13
Temporal involvement			12
Occipital involvement			7
Periventricular			3
Involvement of thalamus, insula or basal ganglia			2
Multifocal			9
Tumour classification (*n*)			
CNS WHO grade 2			2
IDH-mutant			2
1p/19q-codeleted			0
CNS WHO grade 3			6
IDH-mutant			6
1p/19q-codeleted			0
CNS WHO grade 4			27
IDH-mutant			1
MGMT-methylated			13
Clinical score—first surgery			
Charlson comorbidity index (median, range)			3 (2–6)
Deficit pre-op (*n*)			
Motor			7
Sensory			5
Neglect			0
Speech			5
Vision/cranial nerves deficit			5
Vigilance			2
Behavioural changes			2
Headache			6
Seizure			11
Deterioration of general condition			3
Other deficits			0
Tumour volume—first surgery (ml, mean, SEM)		Tumour volume—second surgery (ml, mean, SEM)	
Pre-op T1-CE	28.78 ± 5.25	Post-op T1-CE Post-op T2-nCE	1.2 ± 0.40 0.85 ± 0.22
Pre-op T2-nCE	12.96 ± 3.17		
Post-op T1-CE	7.08 ± 1.53		
Post-op T2-nCE	3.64 ± 0.93		
RANO resect class (*n*)		RANO resect class (*n*)	
1	0	1	10
2A	0	2A	0
2B	2	2B	6
3A	10	3A	7
3B	12	3B	2
4	0	4	0
Residual tumour volume (IDH-mutant tumours, *n*)	9	Residual tumour volume (IDH-mutant tumours, *n*)	9
complete resection	0	complete resection	1
<1 cm^3^	0	<1 cm^3^	3
>1 cm^3^	9	>1 cm^3^	5
Surgical strategy—first surgery (*n*)		Surgical strategy—second surgery (*n*)	
Awake	15	Awake	12
Asleep	17	Asleep	21
Intraoperative neuromonitoring	31	Intraoperative neuromonitoring	33
ISIS Xpert	17	ISIS Xpert	17
C2 Xplore	14	C2 Xplore	16
Fluorescence-guided surgery	28	Fluorescence-guided surgery	26
Clinical score—first surgery		Clinical score—second surgery	
KPS pre first op (median, range)	90 (50–100)	KPS pre second op (median, range)	90 (70–100)
KPS post first op (median, range)	90 (30–100)	KPS post second op (median, range)	90 (40–100)
NIHSS pre first op (median, range)	0 (0–7)	NIHSS pre second op (median, range)	0 (0–3)
NIHSS post first op (median, range)	1.5 (0–12)	NIHSS post second op (median, range)	2 (0–9)
New deficit post-op	10	New deficit post-op	8
Motor	4	Motor	7
Sensory	0	Sensory	0
Neglect	0	Neglect	0
Speech	3	Speech	2
Vision/cranial nerves deficit	4	Vision/cranial nerves deficit	1
Vigilance	0	Vigilance	2
Behavioural changes	0	Behavioural changes	0
Headache	0	Headache	0
Seizure	0	Seizure	0
Deterioration of general condition	1	Deterioration of general condition	0
Other deficits	0	Other deficits	0
Complications of second surgery (*n*)			
Intra-op seizure		1	
Post-op seizure		1	
PAE intra-op		0	
PAE post-op		0	
Cardiac arrest intra-op		0	
Cardiac arrest post-op		0	
Bleeding/infarction		0	
Hydrocephalus		2	
CSF leak		4	
Site infection/delayed wound healing		1	
Clavien–Dindo Classification			
I		7	
II		1	
IIIb		5	
IV		1	
V		0	
Adjuvant therapy for glioblastoma after first diagnosis (*n* = 17)			
TMZ + Rx (Stupp)		14	
TMZ + Rx + CCNU (Herrlinger)		3	
Interval from first surgery to start of radio-chemotherapy (d, median, range)		37.5 (29–66)	

## Data Availability

The data supporting the findings of this study are not publicly available. The ethical approval granted by the local ethics committee does not cover data sharing or transfer to third parties. Therefore, the data cannot be made available to protect patient confidentiality and to comply with ethical and legal requirements.
